# Information and Explanatory Goodness

**DOI:** 10.1007/s10670-023-00687-2

**Published:** 2023-04-20

**Authors:** David H. Glass

**Affiliations:** School of Computing, Ulster University, York St, Belfast, BT15 1ED UK

## Abstract

I propose a qualitative Bayesian account of explanatory goodness that is analogous to the Bayesian account of incremental confirmation. This is achieved by means of a complexity criterion according to which an explanation *h* is good if the reduction in the complexity of the explanandum *e* brought about by *h* (the explanatory gain) is greater than the additional complexity introduced by *h* in the context of *e* (the explanatory cost). To illustrate the account, I apply it in the context of ad hoc hypotheses.

## Introduction

Given an explanatory hypothesis *h* and an explanandum *e*, a number of probabilistic measures of explanatory power (Popper, 1959; Good, 1960; Schupbach & Sprenger, 2011; Crupi & Tentori, 2012), denoted ${\mathcal {E}}(e,h)$, satisfy the following condition:

1 $$\begin{aligned} {\mathcal {E}}(e,h) \gtreqless 0 \;\; \text {if and only if} \;\; P(e|h) \gtreqless P(e), \end{aligned}$$

where *P* represents a probability function.^1^ This provides a *qualitative* account of explanatory power that directly corresponds to the standard Bayesian account of incremental confirmation so that the explanatory power of hypothesis *h* with respect to explanandum *e* is positive if and only if *e* confirms *h*, or equivalently, *h* confirms *e*.

Good (1968) drew attention to an important distinction between explanatory power in the weak sense (weak explanatory power) and the strong sense (strong explanatory power) and noted that ‘the double meaning of “explanatory power” has previously been overlooked’ (Good, 1968, p. 124). By weak explanatory power, he meant that the explanatory power of a hypothesis *h* is ‘unaffected by cluttering up [*h*] with irrelevancies’, while strong explanatory power ‘*is* affected by the cluttering’ (Good, 1968, p. 123). Suppose $h_2$ is probabilistically independent of *e*, $h_1$ and their conjunction, then the degree of weak explanatory power of $h_1$ with respect to *e* is unaffected by the addition of the irrelevant hypothesis, $h_2$, so that ${\mathcal {E}}(e,h_1 \wedge h_2) = {\mathcal {E}}(e,h_1)$. By contrast, strong explanatory power, denoted ${\mathcal {E}}_S(e,h)$, is negatively affected by this cluttering so that in general ${\mathcal {E}}_S(e,h_1 \wedge h_2) < {\mathcal {E}}_S(e,h_1)$. In light of this distinction, all the measures of explanatory power cited at the start of the paper and satisfying condition (1) can be classified as weak measures.^2^

More generally, we can say that weak explanatory power is concerned with how well a hypothesis would explain the explanandum *if the hypothesis were true*. In this context, the connection with confirmation is relevant because the measures of weak explanatory power are also measures of the degree to which *h* confirms *e*. Strong explanatory power, however, is affected by *how likely the hypothesis is to be true in the first place*. The general idea is that strong explanatory power takes into account both the weak explanatory power and the prior probability of the hypothesis and so, for example, if two hypotheses had the same weak explanatory power with respect to an explanandum, the hypothesis with the higher prior probability would have greater strong explanatory power.

The idea in this paper is then to approach explanatory goodness along similar lines to strong explanatory power. Weak explanatory power, while an important concept, is not appropriate for this task because, for example, an ad hoc hypothesis or conspiracy theory with very low prior probability could nevertheless have a positive degree of weak explanatory power (in fact, ad hoc hypotheses are typically designed to do exactly that). In order to make a better assessment of explanatory goodness, the prior probability/complexity of the hypothesis needs to be taken into account along with its weak explanatory power. As discussed above, this is the idea behind strong explanatory power and following Good, I will assume that probability and complexity are inversely related.^3^ While the notions of explanatory goodness and strong explanatory power are very closely related, they are not quite the same since a key issue for the account of explanatory goodness proposed here concerns the conditions under which a hypothesis would provide a good explanation to at least some extent. The goal is to address this point by providing a qualitative account that identifies when a measure of explanatory goodness for an explanation of *e* by *h* is positive/zero/negative. As such, explanatory goodness involves a more specific requirement than strong explanatory power. I will return to the relationship between explanatory goodness and strong explanatory power in Sect. 4.

There are several reasons for seeking a qualitative account of explanatory goodness. Consider Bayesian measures of incremental confirmation by way of comparison. While there are many different measures of confirmation, all of them satisfy the same qualitative account. Hence, those who disagree about how to quantify confirmation, nevertheless agree on the more fundamental principle of what constitutes a measure of confirmation. Similarly, while different measures of explanatory goodness could be proposed, it is arguably more important to obtain clarity on the fundamental idea underlying a probabilistic account of explanatory goodness. Such an account could help to clarify the logic of explanatory reasoning similar to the way in which the Bayesian account of confirmation helps to clarify the logic of inductive reasoning. This could be relevant in the context of inference to the best explanation (IBE), which seems to require an account of explanatory goodness. As Lipton points out, an inference should only be made to the best explanation if it is sufficiently good (Lipton, 2004, p. 154). A qualitative account might help to specify when this is the case or, alternatively, when none of the explanations available is sufficiently good. Another motivation for a qualitative account is that it could provide a first step to obtaining a quantitative account. In fact, I will argue in Sect. 4 that the proposed account helps to define a plausible quantitative account by identifying a particular instance of a measure proposed by Good (1968). As such, the current work plays a key role as part of a wider project to propose a measure of explanatory goodness. Such a project involves defending Good’s general approach which results in a class of strong measures, but does not resolve the qualitative question. By addressing the qualitative question, however, a specific measure of explanatory goodness can then be identified.

## Explanatory Gain and Explanatory Cost

Following Good (1960; 1966; 1968), I will make use of a standard approach to semantic information according to which the information of a proposition *h*, which Good also equates with its complexity, is given by (Bar-Hillel & Carnap, 1953):

2 $$\begin{aligned} \text{ Inf }(h) = - \text{ log } P(h) \end{aligned}$$

and this also applies to the information of *h* conditional on *e*:

3 $$\begin{aligned} \text{ Inf }(h|e) = - \text{ log } P(h|e). \end{aligned}$$

Good also defines the information concerning *h* provided by *e* to be:

4 $$\begin{aligned} \text{ Inf }(h,e) = \text{ log } \left[ \frac{P(e|h)}{P(e)} \right] , \end{aligned}$$

which is Good’s preferred measure of weak explanatory power (see also McGrew, 2003) and again this can also be applied conditional on another proposition, *g* say, to give $\text{ Inf }(h,e|g) = \text{ log } \left[ P(e|h,g)/P(e|g) \right]$. Hence, Good identifies the degree to which *h* weakly explains *e* with the information concerning *h* provided by *e* or equivalently the information concerning *e* provided by *h*. $\text{ Inf }(h,e)$ can also be expressed as:

5 $$\begin{aligned} \text{ Inf }(h,e) = \text{ log } \left[ \frac{P(e|h)}{P(e)} \right] = \text{ Inf }(e) - \text{ Inf }(e|h), \end{aligned}$$

and so if $\text{ Inf }(e)$ is greater than $\text{ Inf }(e|h)$ it represents the difference between the complexity of *e* given only background knowledge and its complexity conditioned on *h* and in this sense we can also think of it as the reduction in complexity of *e* brought about *h*. More informally, we could think of it in terms of the reduction of surprise in *e* brought about by *h*. Note from Eq. (1) that the requirement for weak measures of explanatory power to be positive is equivalent to the requirement that the hypothesis in question reduce the complexity of the explanandum.

Let us now consider the conditions under which a measure of explanatory goodness should be positive (or negative or zero). Central to the approach here is the identification of two factors, which I shall call the *explanatory gain* and the *explanatory cost*. The key proposal in the paper is then to say that for the degree of explanatory goodness to be positive (zero/negative), the explanatory gain should be greater (equal/less) than the explanatory cost. From the discussion in the introduction, it makes sense that explanatory gain should be related to weak explanatory power in some way since it is a positive factor in explanatory goodness. Similarly, explanatory cost should be related to the improbability/complexity of the hypothesis since it is a negative factor. The challenge is how to quantify these factors more precisely.

### Information and Explanatory Gain

Good’s measure of weak explanatory power, $\text {Inf}(h,e)$, seems ideal for explanatory gain since by introducing a hypothesis *h* to explain *e*, the hope is that *h* will reduce the complexity (or equivalently the surprise) of *e*, and this is exactly what Good’s measure quantifies. As noted already, this quantity can be expressed as the information concerning *e* provided by *h*. In other words, it is how informative *h* is about *e*. Adopting this measure of explanatory gain means that the resulting qualitative account of explanatory goodness will then have the qualitative account of weak explanatory power presented in Eq. (1) as a special case. To see this, note that the condition in Eq. (1) can equivalently be stated as:

6 $$\begin{aligned} {\mathcal {E}}(e,h) \gtreqless 0 \;\; \text {if and only if} \;\; \text {Inf}(h,e) \gtreqless 0, \end{aligned}$$

provided $P(e) > 0$. And this is precisely the condition we get if we a) use $\text {Inf}(h,e)$ for explanatory gain, b) specify the degree of explanatory goodness to be positive (zero/negative) when the explanatory gain is greater (equal/less) than the explanatory cost, and c) apply this approach in the special case where the explanatory cost is zero.

As noted earlier, various measures of explanatory power can be classified as weak measures, so why use Good’s preferred measure for explanatory gain? Consider the measures proposed by Schupbach and Sprenger (2011):

7 $$\begin{aligned} {\mathcal {E}}_{SS}(e,h) = \frac{P(h\vert e)-P(h\vert \lnot e)}{P(h\vert e)+P(h\vert \lnot e)} \end{aligned}$$

and Crupi and Tentori (2012):

8 $$\begin{aligned} {\mathcal {E}}_{CT}(e,h) = \left\{ \begin{array}{ll} \dfrac{P(e\vert h)-P(e)}{1-P(e)} \text{ if } P(e\vert h) \ge P(e)\\ \dfrac{P(e\vert h)-P(e)}{P(e)} \text{ if } P(e\vert h) < P(e).\\ \end{array} \right. \end{aligned}$$

A common feature of these measures is that they take on their maximum value of one when *h* entails *e*, assuming that $P(e) < 1$. As such, they can be thought of as quantifying the degree to which *h* entails *e*.^4^ While this may be appropriate for a certain conception of weak explanatory power, arguably it presents a problem as an account of explanatory gain. Suppose *h* entails *e* and that this gives rise to a certain degree of explanatory gain. However, let us further suppose that there is also significant explanatory cost associated with *h* due to its complexity (though we have yet to consider how the cost should be quantified) and that the gain is insufficient to outweigh this cost. It would be reasonable to suppose that *h* could enhance its explanatory gain (and potentially outweigh the cost) by explaining additional evidence, $e^\dagger$. Suppose then that we consider the explanatory gain of *h* with respect to $e\wedge e^\dagger$. The difficulty is that the gain in this case cannot be greater than it is for *e* if the gain is quantified by ${\mathcal {E}}_{SS}$ or ${\mathcal {E}}_{CT}$, and this is so even if *h* also entails $e^\dagger$.^5^ By contrast, Good’s weak measure is additive so that ${\mathcal {E}}(e\wedge e^\dagger ,h) = {\mathcal {E}}(e,h) + {\mathcal {E}}(e^\dagger ,h|e)$, where ${\mathcal {E}}(e^\dagger ,h|e)$ is just the degree to which *h* weakly explains $e^\dagger$ conditional on *e*, and hence if Good’s measure is used, then the explanatory gain of *h* can be greater for $e\wedge e^\dagger$ than it is for *e*.

A further point is that the explanatory gain and cost need to be quantified in a way that enables an appropriate comparison between them. As already noted, Good’s weak measure can be expressed in straightforward information theoretic terms as the information *h* provides about *e* and, as we shall see in Sect. 3, this can be compared with explanatory cost.^6^ An alternative strategy would be to use the main rival measure of semantic information which for *h* is given by (Bar-Hillel & Carnap, 1953)

9 $$\begin{aligned} \text{ Inf}_2\;(h) = 1 - P(h). \end{aligned}$$

Analogous to Eq. (5), we could then define an alternative measure of weak explanatory power as follows:

10 $$\begin{aligned} {\mathcal {E}}_W(e,h) = \text{ Inf}_2(e) - \text{ Inf}_2(e\vert h) = P(e\vert h) - P(e), \end{aligned}$$

which like Good’s measure could be interpreted as the information *h* provides about *e*. However, it is easy to show that ${\mathcal {E}}_W$ is not additive and hence faces a similar difficulty to ${\mathcal {E}}_{SS}$ and ${\mathcal {E}}_{CT}$ as an account of explanatory gain.^7^

While these considerations do not preclude the possibility of exploring the use of some of these measures as part of an overall account of explanatory goodness, they nevertheless provide some reason for adopting Good’s weak measure for explanatory gain in the current context. For further discussion of reasons for preferring Good’s weak measure as part of an account of explanatory goodness, see Glass (2023).

### Information and Explanatory Cost

How should we quantify explanatory cost? As discussed earlier, it should be related in some way to the complexity of the hypothesis introduced, but exactly how the cost should be quantified is less clear. Two possibilities spring to mind: $\text {Inf}(h)$, the complexity of *h* given only background knowledge, and $\text {Inf}(h|e)$, the complexity of *h* given *e*. There is a very straightforward problem with using $\text {Inf}(h)$ to quantify explanatory cost. It is that the explanatory gain could never be greater than the explanatory cost and hence no hypothesis could ever have a positive degree of explanatory goodness. This is because it is always the case that $\text {Inf}(h,e) \le \text {Inf}(h)$. To see this note that:

11 $$\begin{aligned} \text {Inf}(h,e) = \text{ log } \left[ \frac{P(e|h)}{P(e)} \right] = \text{ log } \left[ \frac{P(h|e)}{P(h)} \right] = \text {Inf}(h) - \text {Inf}(h|e), \end{aligned}$$

and since $\text {Inf}(h|e) \ge 0$ it follows that $\text {Inf}(h,e) \le \text {Inf}(h)$. However, if the explanatory cost is represented by $\text {Inf}(h|e)$, then the gain can be greater than the cost.

This suggests that $\text {Inf}(h|e)$ is a more suitable candidate for explanatory cost in the current context, but this pragmatic justification is rather unsatisfying. For example, there might be a concern with the general approach of comparing explanatory gain and cost in this way or that there might be some other way to quantify cost instead of either $\text {Inf}(h|e)$ or $\text {Inf}(h)$. The following discussion is intended to provide further justification for $\text {Inf}(h|e)$.

#### No Cost and Low Cost Explanations

Consider the circumstances in which there would be no explanatory cost. Given that the cost should be related to the complexity of the hypothesis, it seems plausible that for an explanandum *e*, there should be no explanatory cost associated with a hypothesis *h* if $\text {Inf}(h) = 0$, which would occur when $P(h) = 1$. However, in this case $\text {Inf}(h|e)$ would also be zero since $P(h|e) = 1$. Nevertheless, $\text {Inf}(h|e)$ could be zero even if $\text {Inf}(h) > 0$. In such a case, should we consider the explanatory cost to be zero? Suppose two mutually exclusive and exhaustive hypotheses are being considered for a coin: one that it is a fair coin ($h_1$), the other that it is double headed ($h_2$). Suppose further that both of these hypotheses are assigned equal prior probabilities based on background knowledge. The coin is then tossed and lands tails (*e*). Clearly, $P(h_1|e) = 1$ and so $\text {Inf}(h_1|e) = 0$, while $P(h_1) = 0.5$ and so $\text {Inf}(h_1) = \text {log} \; 2$. Since the explanandum *e* entails $h_1$ this seems like a clear case where there is no explanatory cost involved with $h_1$; given *e*, we get $h_1$ for free. Hence, $\text {Inf}(h|e)$ provides a suitable measure of explanatory cost in this case. It is also worth noting that the explanatory gain for $h_1$ is positive ($\text {Inf}(h_1,e) = \text {log} \; 2$), so the explanatory goodness should be positive in this case.^8^

A similar point applies to cases where there is a low explanatory cost. $\text {Inf}(h|e)$ could be low even if $\text {Inf}(h)$ is high. Should we consider the cost to be low in this case? Suppose the prior probability of Smith’s guilt (*h*) is low in a murder investigation so that $\text {Inf}(h)$ is high. However, on the basis of DNA evidence (*e*), the posterior probability of Smith’s guilt is high, so that $\text {Inf}(h|e)$ is low. In the limiting case where $\text {Inf}(h|e) = 0$, we have seen that there is no cost associated with *h*. In the current case, *h* does not follow from *e* with probability one, but it still follows with high probability, so it seems reasonable to conclude that the cost associated with *h* is low.^9^ If this is right, then as in the no cost case, $\text {Inf}(h|e)$ provides a plausible way to quantify explanatory cost.

#### Additional Complexity Introduced by *h*

Another reason why $\text {Inf}(h|e)$ is appropriate as a way to quantify the explanatory cost is because it represents the complexity that goes beyond that already provided by *e*. To see this, recall from Eq. (11) that $\text {Inf}(h|e)$ can be expressed as $\text {Inf}(h) - \text {Inf}(h,e)$, so it is the complexity of *h* minus the information or complexity about *h* provided by *e*. Hence, $\text {Inf}(h|e)$ represents the *additional* complexity introduced by *h* in the context of *e*. It is very plausible to think that it is the additional complexity that is relevant to the explanatory cost since if we were to use $\text {Inf}(h)$ it would, in effect, double count the complexity of *h* already provided by *e*. In fact, thinking of it in this way helps to diagnose what would be wrong with using $\text {Inf}(h)$ in the no and low cost cases. Consider the no cost case where $\text {Inf}(h|e) = 0$, but $\text {Inf}(h) > 0$. Using $\text {Inf}(h|e)$ to quantify explanatory cost takes into account the fact that *h* introduces no additional complexity beyond that provided by *e*.

Having identified the relevant quantities for both explanatory gain and explanatory cost, we are now in a position to consider the complexity criterion for explanatory goodness.

## The Complexity Criterion

In light of the foregoing discussion, I propose the following criterion for explanatory goodness:

**Complexity criterion for explanatory goodness**: If ${\mathcal {E}}_G(e,h)$ is a measure of explanatory goodness of an explanatory hypothesis *h* for explanandum *e* then:^10^

$$\begin{aligned} {\mathcal {E}}_G(e,h) \gtreqless 0 \;\; \text {if and only if} \;\; \text {Inf}(h,e) \gtreqless \text {Inf}(h|e). \end{aligned}$$

In terms of information, the complexity criterion for explanatory goodness is that an explanation is good to at least some extent if the information concerning *e* provided by *h* is greater than the information content of *h* given *e*. Or in terms of complexity, an explanation is good if the reduction in complexity of *e* brought about by *h* is greater than the complexity of *h* given *e*, which is the additional complexity introduced by *h* in the context of *e*. Informally, we could say that for an explanation to be good to some degree, it must pay its way in terms of complexity.^11^

Note that since $\text {Inf}(h|e) \ge 0$, a necessary, but not sufficient, requirement for explanatory goodness to be positive is that $\text {Inf}(h,e) > 0$. Hence, its degree of explanatory gain must also be positive. It may be that in some cases, all of the explanations available have a negative degree of explanatory goodness. In such cases, it could be that the best explanation actually has a negative degree of explanatory gain as well and so is negatively related to the explanandum. This could occur, for example, if it had a much higher posterior probability than any of the other explanations. Nevertheless, having a negative degree of explanatory gain, there would be something deficient about this explanation according to the complexity criterion.

Related to this, just because an explanation has a negative degree of explanatory goodness in the sense defined above, this does not necessarily mean that it has no merit to it. As just indicated, it could be the best explanation available, or it could have a high degree of explanatory gain. However, the point is that the reduction of complexity of *e* brought about *h* is not sufficient to outweigh the additional complexity introduced by *h*. Nevertheless, there is the possibility that if further evidence became available that *h* could also explain, then its explanatory goodness for all the evidence could become positive. Hence, if an explanatory hypothesis has at least some redeeming features, it might be worthy of consideration and further evidence could be sought.

An alternative strategy would be to use the rival account of semantic information in Eq. (9) and hence the measure of explanatory gain in Eq. (10). Adopting this approach, explanatory cost would be $1-P(h|e)$ and hence the relevant condition for the complexity criterion would become $P(e|h)-P(e) \gtreqless 1-P(h|e)$. While this approach also has some plausibility, it encounters a potential problem due to the fact that it is not additive as discussed in Sect. 2.1. If it is used as an account of explanatory goodness, this can result in cases where a hypothesis has a positive degree of explanatory goodness for *e*, say, provides explanatory gain for additional evidence $e^\dagger$, which in turn further confirms *h* and hence reduces the explanatory cost of *h*, and yet *h* has a negative degree of explanatory goodness for $e \wedge e^\dagger$.

With the complexity criterion in place, let us revisit the earlier discussion about $\text {Inf}(h|e)$ and $\text {Inf}(h)$. Although reasons were given for preferring the former to quantify explanatory cost, it is clear that the latter expression, which represents the complexity of *h* on background knowledge, is still relevant. The requirement for explanatory goodness to be positive, $\text {Inf}(h,e) > \text {Inf}(h|e)$, is equivalent to $2\,\text {Inf}(h,e) > \text {Inf}(h)$. So in general, hypotheses with high values of $\text {Inf}(h)$ need to have greater explanatory gain if they are to provide good explanations. Nonetheless, $\text {Inf}(h)$ does not provide a suitable explication of explanatory cost, which is necessary for the justification of the complexity criterion.

It is worth noting the resemblance between the complexity criterion and the standard Bayesian account of incremental confirmation (see also expression (1)), according to which a measure of the degree to which *e* confirms *h* is positive (zero, negative) if and only if $P(e|h) \gtreqless P(e)$ or, equivalently if and only if $\text {Inf}(h,e) \gtreqless 0$. Thus, the complexity criterion can be seen as providing a qualitative Bayesian characterization of explanatory goodness that is analogous to the qualitative characterization of confirmation. Related to this point, it would be possible to construct various measures of explanatory goodness that are not ordinally equivalent as is the case with measures of confirmation. However, provided they satisfy the complexity criterion, such measures will agree about when explanatory goodness should be positive, just as measures of confirmation agree about whether evidence confirms a given hypothesis.

A possible objection to this approach is that any account of explanatory goodness should include a detailed analysis of explanatory virtues. Advocates of measures of (weak) explanatory power have sought to do this and, insofar as they have been successful, their results also apply to the current approach since a good explanation requires a positive degree of weak explanatory power because otherwise the explanatory gain would not be positive.^12^ However, weak explanatory power fails to accommodate the simplicity/complexity of explanations and so condition (1) would not provide a satisfactory account of explanatory goodness in a general sense. By contrast, since the simplicity/complexity is accommodated within the complexity criterion in terms of the explanatory cost, arguably it offers a more plausible account of explanatory goodness. Of course, whether it fully captures explanatory goodness is a question that would merit further investigation. Can explanatory gain and explanatory cost do justice to all the explanatory virtues? It must be acknowledged that there could be limitations arising from the fact that the account does not capture the nature of the explanatory relation itself and, related to this, it would be worth exploring how causality could be incorporated within the account. Nevertheless, while an in-depth treatment of the explanatory virtues is beyond the scope of this paper, by incorporating the advantages of weak explanatory power along with simplicity/complexity, the current approach can be proposed as capturing key aspects of explanatory goodness (for further discussion, see Glass, 2023).

I will now illustrate how the approach can be applied to ad hoc hypotheses. Consider the case of Dianetics discussed by Howson and Urbach (1993). L. Ron Hubbard, the inventor of Dianetics, claimed that a young woman who had undergone therapy represented a triumphant success. In 1950,

[Hubbard] exhibited this person to a large audience, claiming that she had a ‘full and perfect recall of every moment of her life’. But questions from the floor (‘What did you have for breakfast on October 3, 1942?’; ‘What color is Mr. Hubbard’s tie?’, and the like) soon demonstrated that the hapless young woman had a most imperfect memory. Hubbard accounted for this ... by saying that when the woman first appeared on the stage and was asked to come forward ‘now’, the word ‘now’ had frozen her in ‘present time’ and paralysed her ability to recall the past. (Howson and Urbach 1993, p. 148)

Let *h* represent the conjunction of the defining propositional tenets of Dianetics, *a* the claim that the word ‘now’ had frozen the woman in ‘present time’, and *e* her failure to answer the questions correctly. This seems like a clear case where $h \wedge a$ is ad hoc (in a negative sense)^13^ and, if so, we would expect it to be a bad explanation to some degree, i.e. have a negative degree of explanatory goodness. We can grant that it has some positive explanatory gain since *a* ensures that $h \wedge a$ entails *e*. This means that weak measures would assign a positive degree of explanatory power to $h \wedge a$. However, the gain is not that great since *e* is not very surprising at all. An important factor here is that $P(e|\lnot h$) is not too low since the woman’s failure to answer the question is not surprising at all if Dianetics is false. On the other hand, *a* itself is highly improbable and hence $h \wedge a$ is highly improbable too. More pertinently, $h \wedge a$ is highly improbable given *e* and so the hypothesis introduces a lot of additional complexity in the context of *e*. Hence, the explanatory cost is very high. Along with low explanatory gain, this ensures that $h \wedge a$ is a bad explanation.

Now consider the discovery of the planet Neptune. It is well-known that a new planet was proposed (*a*) to account for the observations of the orbit of Uranus (*e*), which were found to be in conflict with Newtonian theory (*h*) assuming the planets known at that time. The success of this strategy led to the discovery of Neptune soon afterwards. This seems like a clear case where $h \wedge a$ is not ad hoc (in a negative sense). As in the previous case, there is positive explanatory gain since *a* ensures that $h \wedge a$ entails *e*. However, in this case the explanatory gain is very high since *e* was very surprising. In contrast to the Dianetics case, $P(e|\lnot h)$ would also have been low since there was no reason to expect *e* on the assumption that Newtonian theory was false.^14^ While *a*, and hence $h \wedge a$, was very improbable, crucially $h \wedge a$ was much less improbable given *e* and so arguably $P(h \wedge a|e)$ would not have been too low. Too see why, note that $P(h \wedge a|e) = P(a|h \wedge e) \times P(h|e)$. While there were some alternative proposals consistent with *h*, plausibly $P(a|h \wedge e)$ was quite high. And given the high prior for *h* (based on earlier evidence) and the fact that $P(e|\lnot h)$ was low, there is no reason to think that *P*(*h*|*e*) was very low even though *e* may well have disconfirmed *h* to some extent.^15^ Hence, the additional complexity in the context of *e* is not so high since it is just log$[1/P(h \wedge a|e)]$. Along with a very high explanatory gain, this ensures that $h \wedge a$ is a good explanation.

There is much more that could be said about ad hoc hypotheses and a detailed treatment would require exploring how the current approach relates to the accounts provided by Strevens (2001) and McGrew (2014). Nevertheless, this brief discussion suggests that the proposed approach is able to distinguish between good and bad explanations appropriately.

## Towards a Quantitative Account

Good (1968) defined and presented a detailed defence of the following measure of strong explanatory power:

12 $$\begin{aligned} {\mathcal {E}}_{Good}(e,h)&= \text{ log } \left[ \frac{P(e|h)\cdot P(h)^\gamma }{P(e)} \right] \nonumber \\&= \text {Inf}(h,e) - \gamma \text {Inf}(h)\nonumber \\&= (1 - \gamma ) \text {Inf}(h,e) - \gamma \text {Inf}(h|e). \end{aligned}$$

where $0< \gamma < 1$ is a constant. Note that it can be expressed in terms of explanatory gain and explanatory cost. Returning to our earlier discussion about how to quantify cost, it is instructive to consider Good’s discussion of what would constitute a ‘full explanation’. He claims that ‘ideally we want $[P(e|h)] = 1$ *and* $[P(h)] = 1$’ (1968, p. 131). However, when $P(h) = 1$, not only does $\text {Inf}(h) = 0$, but $\text {Inf}(h,e) = 0$ and so the strong explanatory power according to ${\mathcal {E}}_{Good}$ is in fact zero. So requiring that $P(h) = 1$ and hence $\text {Inf}(h) = 0$ is not an appropriate requirement for a ‘full explanation’. According to Good’s measure, a more appropriate requirement is that $\text {Inf}(h|e) = 0$ as can be seen from the last line of Eq. (12). This in turn requires that $P(h|e) = 1$. A further requirement would be that $P(e|h) = 1$ (to ensure $\text {Inf}(h,e)$ is as large as possible) for *h* to be the best possible explanation for a given *e*. So for a ‘full explanation’, the requirement is that $P(e|h) = 1$ and $P(h|e) = 1$, but $P(h) < 1$. Or equivalently, it is that the explanatory gain is as large as possible for *e* and that explanatory cost is zero. Hence, the conditions for a full explanation provide a further reason for thinking that the proposed explications of explanatory gain and explanatory cost are appropriate.

From Eq. (12), we can see that ${\mathcal {E}}_{Good}(e,h) > 0$ if and only if $(1 - \gamma ) \text {Inf}(h,e) > \gamma \text {Inf}(h|e)$ and hence if ${\mathcal {E}}_{Good}$ is to satisfy the complexity criterion, $\gamma$ must be ${1}/{2}$. Interestingly, with this value, Good’s measure is essentially just explanatory gain minus explanatory cost, which provides a straightforward quantitative measure of explanatory goodness. Other values of $\gamma$ would result in measures of strong explanatory power that penalize hypotheses for their complexity, but would not constitute measures of explanatory goodness. Good thought that setting $\gamma = {1}/{2}$ provides the simplest explicatum since it gives equal weighting to (weak) explanatory power and what he calls ‘the avoidance of “clutter”’ [p. 130], but arguably the reasons given here provide further justification for this claim.

An alternative to Good’s measure could be obtained by dividing the relevant factors to give $\text {Inf}(h,e)/\text {Inf}(h|e)$. This measure, which we can call the ratio measure, satisfies a slightly modified version of the complexity criterion since it is greater than one (rather than zero) when $\text {Inf}(h,e) > \text {Inf}(h|e)$. Nevertheless, there are reasons for preferring Good’s measure with ($\gamma = {1}/{2}$) based on limiting cases. For example, consider the case where *h* provides no explanatory gain for *e*, i.e. $P(e|h) = P(e)$. The ratio measure is zero in this limit, whereas Good’s measure is $-\text {Inf}(h|e)$ which represents the explanatory cost and so it preserves important information that can discriminate between different explanations. Similarly, consider the limiting case where there is no explanatory cost, i.e. where $P(h|e) = 1$. In this case, the ratio measure is undefined, whereas Good’s measure is just the explanatory gain, and so once again it is able to discriminate between different explanations.

In this section, I have not attempted to provide a detailed justification of Good’s strong measure as a measure of explanatory goodness, but just that if one adopts his approach, the complexity criterion provides a reason for setting $\gamma$ to 1/2. A more general justification of this measure would require exploring a range of properties that should be satisfied and would take us beyond the scope of this paper. For a detailed defence of Good’s measure as a quantitative measure of explanatory goodness along with a detailed comparison of relevant properties of weak and strong measures, see Glass (2023).

## Conclusion

While a number of measures of explanatory power have their merits, an account of explanatory goodness needs to take into account the improbability/complexity of explanatory hypotheses. To address this, I have proposed a qualitative Bayesian account based on a comparison of explanatory gain and explanatory cost. It would be interesting to explore what implications this might have for debates about inference to the best explanation and its relationship to Bayesianism, as well as for explanatory reasoning more generally.

## Footnotes

1 Probabilities are assumed to be conditional on relevant background knowledge, but this is suppressed in the notation for simplicity.

2 These measures satisfy the principle of positive relevance which states that ${\mathcal {E}}(e,h_1) \gtreqless {\mathcal {E}}(e,h_2)$ if and only if $P(e|h_1) \gtreqless P(e|h_2)$ (Cohen, 2016) From this it follows, that ${\mathcal {E}}(e,h_1 \wedge h_2) = {\mathcal {E}}(e,h_1)$ in the case of an irrelevant hypothesis $h_2$.

3 Intuitively, this makes sense for the more ‘cluttered’ hypothesis $h_1 \wedge h_2$ since its probability is less than or equal to the probability of $h_1$. More generally it is based on a standard approach to semantic information (see Sect. 2).

4 For discussion of the ${\mathcal {E}}_{CT}$ as a measure of partial entailment, see Crupi and Tentori (2013, 2014)

5 As we shall see, in the proposed account the additional evidence could also reduce the cost, but it seems reasonable that it should also enhance the gain.

6 Of course, this is not to deny that the other measures can be expressed in information theoretic terms. Crupi and Tentori (2014) provide a very helpful discussion of the relationship between information and confirmation. As they show, their preferred measure of confirmation given in Eq. (8) has a straightforward interpretation in terms of the alternative account of information in Eq. (9). In the terminology used here, the measure ${\mathcal {E}}_{CT}$ could be expressed as the information *h* provides about *e* divided by the information content of *e* in the case where $P(e|h) > P(e)$.

7 ${\mathcal {E}}_W$ differs from ${\mathcal {E}}_{SS}$ and ${\mathcal {E}}_{CT}$ in that in some cases where *h* entails *e*, ${\mathcal {E}}_W(e\wedge e^\dagger ,h)$ is greater than ${\mathcal {E}}_W(e,h)$. For example, this would be the case if *h* also entails $e^\dagger$ provided $P(e^\dagger |e) < 1$.

8 Note also that if $\text {Inf}(h) = 0$, then the explanatory gain must also be zero.

9 In such scenarios, the low cost will be accompanied by a high explanatory gain since from Bayes’ theorem a high value of *P*(*e*|*h*)/*P*(*e*) (and so a high explanatory gain) is needed to convert a low prior for *h* to a high posterior, but in other cases a high gain can be accompanied by a high cost.

10 Note that a measure of explanatory goodness is not intended as an account of what constitutes an explanation, but rather presupposes such an account and is only intended to quantify the strength of the relationship. Hence, it only applies when *h* is an explanatory hypothesis for *e* and not to purely probabilistic correlations, for example. Also note that *P*(*e*) and *P*(*h*|*e*) are assumed to be non-zero. The requirement that $\text {Inf}(h,e) \gtreqless \text {Inf}(h|e)$ is equivalent to $P(e|h)/P(e) \gtreqless 1/P(h|e)$.

11 It is important to emphasize that the criterion does not require that the introduction of *h* in addition to *e* reduce the overall complexity compared to that of *e* on its own. Clearly, that would be impossible since $\text {Inf}(e \wedge h) = \text {Inf}(e) + \text {Inf}(h|e) \ge \text {Inf}(e)$. Rather, the criterion only requires that the explanatory gain be greater than the explanatory cost.

12 See, for example, Myrvold’s (2003) account of unification and McGrew’s (2016) account of diverse evidence.

13 Sometimes it is just the auxiliary hypothesis *a* that is referred to as ad hoc and sometimes it is the conjunction $h \wedge a$. For our purposes, it is the explanatory goodness of $h \wedge a$ that needs to be considered. Also, ‘ad hoc’ can be used in a neutral sense to refer to cases such as the two considered where there is no independent evidence to support *a*, but if used in this way, we need to be able to distinguish negative cases like the Dianetics example from positive cases like the Uranus example.

14 This is not to deny the possibility – either in this or other cases – that there could be a hypothesis $h'$ that is incompatible with *h* and which makes *e* probable. However, unless such a hypothesis were itself probable given the falsity of *h*, this would provide no reason to think that $P(e|\lnot h)$ is high. Nevertheless, as a referee has pointed out, such a hypothesis could be just as probable given *e* as $h \wedge a$ without making $h \wedge a$ ad hoc.

15 Using Bayes’ theorem, $P(h \wedge a|e) = \frac{P(e|h \wedge a)}{P(e)}P(h \wedge a)$ and so the low prior for $h \wedge a$ would be boosted by the high value of $P(e|h \wedge a)/P(e)$ corresponding to the explanatory gain.
